# Non-canonical EphA2 activation underpins PTEN-mediated metastatic migration and poor clinical outcome in prostate cancer

**DOI:** 10.1038/s41416-022-01914-3

**Published:** 2022-07-22

**Authors:** Ashwin Sachdeva, Claire A. Hart, Kyungmin Kim, Thomas Tawadros, Pedro Oliveira, Jonathan Shanks, Mick Brown, Noel Clarke

**Affiliations:** 1 Genito Urinary Cancer Research Group, Division of Cancer Sciences, Faculty of Biology, Medicine & Health, The University of Manchester and FASTMAN, Prostate Cancer UK & Movember Centre of Excellence, Manchester, UK; 2 Department of Surgery, The Christie NHS Foundation Trust, Manchester, UK; 3 Department of Pathology, The Christie NHS Foundation Trust, Manchester, UK; 4 Department of Urology, Salford Royal NHS Foundation Trust, Salford, UK

**Keywords:** Prostate cancer, Bone metastases

## Abstract

**Background:**

The key process of mesenchymal to amoeboid transition (MAT), which enables prostate cancer (PCa) transendothelial migration and subsequent development of metastases in red bone marrow stroma, is driven by phosphorylation of EphA2^S897^ by pAkt, which is induced by the omega-6 polyunsaturated fatty acid arachidonic acid. Here we investigate the influence of EphA2 signalling in PCa progression and long-term survival.

**Methods:**

The mechanisms underpinning metastatic biopotential of altered EphA2 signalling in relation to PTEN status were assessed in vitro using canonical (EphA2^D739N^) and non-canonical (EphA2^S897G^) PC3-M mutants, interrogation of publicly available PTEN-stratified databases and clinical validation using a PCa TMA (*n* = 177) with long-term follow-up data. Spatial heterogeneity of EphA2 was assessed using a radical prostatectomy cohort (*n* = 67).

**Results:**

Non-canonical EphA2 signalling via pEphA2^S897^ is required for PCa transendothelial invasion of bone marrow endothelium. High expression of EphA2 or pEphA2^S897^ in a PTEN^low^ background is associated with poor overall survival. Expression of EphA2, pEphA2^S897^ and the associated MAT marker pMLC2 are spatially regulated with the highest levels found within lesion areas within 500 µm of the prostate margin.

**Conclusion:**

EphA2 MAT-related signalling confers transendothelial invasion. This is associated with a substantially worse prognosis in PTEN-deficient PCa.

## Introduction

PCa preferentially metastasises to the bone marrow of the axial skeleton causing major morbidity and mortality [1, 2]. Recent advances in understanding the mechanism of this site-directed PCa metastasis [3–5], coupled with epidemiological studies [6–9] have highlighted the role of specific lipids in the metastatic process. In particular, the omega-6 polyunsaturated fatty acid (ω-6 PUFA) arachidonic acid (AA), found in bone marrow adipocytes [10–12], has been shown to promote an aggressive migratory phenotype [13–15] by driving a morphological switch from epithelial to mesenchymal transition (EMT) to mesenchymal to amoeboid transition (MAT). This process is essential for transendothelial invasion across the blood-bone-endothelial barrier [16] and entry into the bone marrow stroma. Characterisation of the molecular mechanism behind AA-induced amoeboid transformation has shown that non-canonical ligand-independent EphA2 signalling [17] is critical for AA-induced PCa endothelial transmigration [16, 18] and therefore it is of critical importance in the development of metastatic spread, the lethal form of prostate cancer.

EphA2 belongs to the largest family of tyrosine kinase receptors (RTK). Ephrin receptors (Eph), through interaction with their membrane-bound ephrin ligands (EFN), induce contact-dependent bidirectional signalling that regulates diverse migrational processes including reduced cell polarity, cell-cell adhesion and motility (reviewed in Cioce and Fazio [19]). Eph receptors have been implicated in tumour formation and progression, with EphA2 being the most frequently perturbed Eph receptor associated with cancer development and progression [20], including both prostate [21] and breast [22] cancer. However, it has been shown to have dual and conflicting roles, acting as both a tumour suppressor and driver. This paradoxical behaviour is dependent on the manner of activation of the EphA2 receptor. Canonical EphA2 signalling initiated through the binding of an ephrin ligand e.g. ephrin-A1 (EFNA1), to EphA2 induces autophosphorylation and induction of EphA2 tyrosine kinase activity through phosphorylation of tyrosine 772 (pEphA2^Y772^). This phosphorylation inhibits cell proliferation and migration by blocking integrin, Ras/Erk and Rac signalling pathways [16, 18, 23–25]. EphA2 signalling has also been shown to induce migratory behaviour in epithelial cells, thereby promoting tumour progression by driving invasion and metastasis. This process involves a non-canonical ligand-independent activation pathway, whereby EphA2 is phosphorylated at serine 897 (pEphA2^S897^) by pAkt or ribosomal S6 kinase (RSK) [26, 27].

Constitutively activated Akt is a common feature of PCa due to loss of Phosphatase and Tensin homolog (PTEN). PTEN, which is one of the most common genetic alterations detected in PCa, presents in 10–46% of tumours, promoting tumour progression and proliferation of cells with a metastatic phenotype characterised by increased cell survival and the ability to invade and migrate to secondary sites. Loss of PTEN expression has been shown to correlate with adverse clinical features such as increased Gleason score, extra-prostatic extension and PCa-specific death (reviewed in Jamaspishvili et al. [28]).

Non-canonical EphA2 signalling initiates the Rho/ROCK signalling cascade and downstream phosphorylation of myosin light chain 2 (pMLC2) leading to a morphological switch associated with malignant prostate epithelial cell behaviour. This switch from mesenchymal to amoeboid morphology (MAT), is essential for transendothelial invasion into the bone marrow stroma [16, 18]. The function of non-canonical EphA2 activity as a promoter of cellular metastatic behaviour can be blocked by the addition of the ephrin-A1 ligand [18], with subsequent internalisation and degradation of the receptor. This has led to research evaluating the potential of using the ephrin-A1 ligand as a potential therapeutic target. Unfortunately, the low specificity of EphA2 for the ephrin-A1 ligand has prevented its use [29]. Recently, there has been an increased interest in EphA2 as a therapeutic target with the development of EphA2-specific agonists. Ephrin ligand mimicking peptides [30–32] engineered into multivalent structures have been shown to be potent stimulators of canonical EphA2 activity, with subsequent internalisation and degradation of the receptor, preventing non-canonical EphA2 activity and thereby suppressing tumour progression.

Herein, we show that non-canonical EphA2 activity (pEphA2^S897^) is crucial to prostate cellular invasion in vitro and that its presence in vivo in primary PTEN-deficient PCa identifies patients who have a substantially increased risk of PCa progression and mortality. We also demonstrate the spatial heterogeneity in the expression of PTEN, EphA2 and a marker of MAT, pMLC2, with lesions closest to the prostate margin exhibiting a more aggressive phenotype.

## Materials and methods

### Prostate tissue

In accordance with the Declaration of Helsinki, archival FFPE specimens from men undergoing prostate biopsy or transurethral resection of the prostate at Salford Royal NHS Foundation Trust were collected under MCRC Biobank Ethics 10_NOCL_02, Manchester, UK. These were used to construct a tissue microarray (TMA) comprising multiple tumour and normal-adjacent cores from a total of 270 patients allied to long-term clinical outcome data (>10 years). Archival FFPE specimens were acquired under MCRC Biobank Ethics TMA_NOCL_01 (18_MIBR_02) from 67 men undergoing radical prostatectomy at The Christie NHS Foundation Trust, Manchester, UK. H&E-stained TMA tissue sections were annotated for Gleason Grade Group by a specialist uropathologist blinded to existing clinical data.

### Cell lines

The PC3 prostate cell line was obtained from the European Collection of Authenticated Cell Cultures (ECACC), UK and PC-3M was a gift from Dr Jason Carrol (CRUK Cambridge, UK). Both cell lines were cultured in Ham’s F12, 10% FCS and 2 mM L-glutamine. The PC3-PTEN inducible cell line has been described previously [33]. BMEC cell line [34] was cultured in primary bone marrow culture conditioned medium (Iscove’s Modified Dulbecco’s media/10% FCS/10% horse serum and 5 × 10^−7^ M hydrocortisone) in fibronectin (50 μg/ml) coated flasks. Cultures were grown at 37 °C in a humidified atmosphere of 5% CO_2_ in the air. Cell lines were authenticated and tested for mycoplasma contamination through the CRUK-Manchester Institute Molecular Biology Core Facility. All cells tested negative for mycoplasma contamination.

### Invasion assay

Invasion of seeded epithelial cells across Matrigel and endothelial cell barriers was measured objectively in invasion chambers. FluoroBlok cell culture inserts (8-µm pore size) (Corning, Amsterdam, NL) were coated with Matrigel diluted 1:25 with RPMI w/o phenol red and BMEC cells grown to confluence. These were placed in a 24-well plate containing 1 mL of RPMI/0.1% fatty acid-free bovine serum albumin (FAF BSA) as control (CON) or with arachidonic acid 20 µM (AA) added. Epithelial cells (5 × 10^4^ cells in 0.25 mL of RPMI/0.1% FAF BSA) were seeded onto the top of the inserts after first labelling with DilC16 (3) (Invitrogen, Paisley UK). After 18 h, the cells were read on a FLUOstar OPTIMA bottom reading fluorescence plate reader (BMG Labtech, Aylesbury, UK) at 544/590 nm after being incubated at 37 °C/5% CO_2_. For the real-time invasion assay, the inserts were incubated directly within the plate reader at 37 °C/5% CO_2_ and read at 1-h intervals up to 24 h. Each experiment was carried out in triplicate [35].

### Generation of EphA2 phospho mutants

The EphA2 gene was cloned into a third-generation HIV-derived lentiviral vector under the control of the CMV promoter. Defective canonical (EphA2^D739N^) and non-canonical (EphA2^S897G^) mutants were generated by site-directed mutagenesis and verified by Sanger Sequencing (Supplementary Fig. 1). These mutated versions were then transduced into the PC3-M EphA2^KO^ cell line using a third-generation HIV-derived lentiviral vector under the control of the CMV promoter with expression confirmed by western blot.

### Western blots

Cell lysates for western blot were prepared using SDS buffer (62.5 mM Tris-EDTA, pH6.8, 5% SDS) and total protein content was measured using the BCA protein assay kit (Bio-Rad Laboratories, Hertfordshire, UK). Lysates were resolved on a 4–20% polyacrylamide gel (Pierce/Thermo Fisher, Warrington, UK) and transferred to a Hybond ECl nitrocellulose membrane (Amersham, GE Healthcare, Buckinghamshire, UK). Membranes were blotted overnight at 4 °C with the indicated primary antibodies (Supplementary Table 1) and developed using the HRP substrate western blot detection system (Merck/Millipore, Watford, UK). Blots were visualised with a ChemiDoc Touch Imaging System (Bio-Rad, Hemel Hempstead, UK) and band intensities were analysed using the MacBioPhotonics plugin and Image J software.

### Multiplex immunofluorescence

A detailed description of the materials and methods is presented within the supplementary methodology. In brief, multiplex immunofluorescence labelling was performed on tissue sections (4 µm) dewaxed with heat-induced epitope retrieval (TRIS/EDTA, pH 9.0). Sections were labelled with 1:100 anti-pEphA2^S897^ overnight at 4 °C. Following a PBS wash and 0.3% H_2_O_2_ block, slides were loaded onto a Ventana Discovery Ultra automated IHC/ISH research platform for HRP conjugation and TSA Opal 620 1:100 linkage. This was repeated sequentially for EphA2 (C-3) 4 µg/mL/TSA Opal 570 1:150; PTEN (D4.3) 1:50/TSA Opal 520 1:100; pan-cytokeratin (C11) 1:10,000/TSA Opal 690 1:150 and DAPI. ProLong Gold-mounted slides (Invitrogen, Paisley, UK) were scanned on a Vectra 3 microscope (Akoya Biosciences Inc., Marlborough, MA 01752, USA) at x20 magnification with automated image analysis by InForm v2.4 (Akoya Biosciences), which provided single-cell level counts for pEphA2^S897^, EphA2 and PTEN and stratified into epithelial and stromal compartments. These data were subsequently analysed using RStudio v1.1.423.

The single-cell level cytoplasmic intensity was acquired for each of the markers and normalised against data from adjacent benign tissue regions by calculating Z-scores. For TMA data, a mean Z-score was calculated for each marker for each individual core. As we hypothesised that high EphA2, high pEphA2^S897^ and low PTEN status were likely to impact outcome adversely, for patients with multiple tumour cores, patient status was determined using the core with highest mean EphA2 intensity, highest mean pEphA2^S897^ intensity and lowest PTEN intensity.

pEphA2^S897^, EphA2 and PTEN status were determined at the patient level using the *survminer* package, which classified patients into high and low expression groups using maximal rank statistics. Kaplan–Meier survival curves were generated for overall survival and compared using the log-rank test. Univariate and multivariate analyses were performed using Cox proportional hazard models and group comparisons using generalised linear models using the R package *finalfit*. Covariates used for multivariate analyses included age at diagnosis, PSA at diagnosis, Grade Group, clinical tumour stage and presence of metastatic disease.

### Spatial expression of markers

H&E-stained radical prostatectomy tissue sections were annotated for tumour regions, normal-adjacent regions, and the prostate margin by a specialist uropathologist (PO) to evaluate spatial heterogeneity in marker expression (Supplementary Fig. 2). Regions of interest within tumour regions were labelled as *x*, *y*, or *z*, at 500 µm, 500–1000 µm and 1000–1500 µm from the prostate margin, respectively. Normal-adjacent regions were labelled *n*. Serial sections were subjected to multiplex immunofluorescence labelling PTEN, EphA2, pEphA2^S897^and pMLC2 as described above. Slides were scanned using the Vectra 3 microscope (Akoya Biosciences Inc., Marlborough, MA 01752, USA) at ×20 magnification and images were analysed using the HALO platform v3.0.311.299 (Indica Labs, Albuquerque, USA) for single-cell segmentation with associated spatial co-ordinates. Z-scores for each marker were calculated across the whole cohort at the single-cell level.

### Ephrin signalling interrogation of external datasets

The publicly available prostate cancer datasets TCGA-PRAD PanCancer Atlas (*n* = 494) [36] and MSKCC Prostate Oncogenome Project (*n* = 240) [37] were interrogated within cBioportal using an Ephrin signalling signature (SHC1/GNA11/MAPK1/RRAS/ARHGEF15/EFNA3/EFNA4/EFNA5/SH2D3C/RAPGEF1) [38]. Oncoprints of upregulated Ephrin signalling and Kaplan–Meier disease-free survival analysis for patients with available survival data in TCGA-PRAD (*n* = 334) and MSKCC (*n* = 112) cohorts stratified by either Ephrin signalling alone or in combination with PTEN status were reported.

## Results

### Upregulation of Ephrin signalling correlates with reduced disease-free survival in PTEN-deficient prostate cancer

**Fig. 1:**
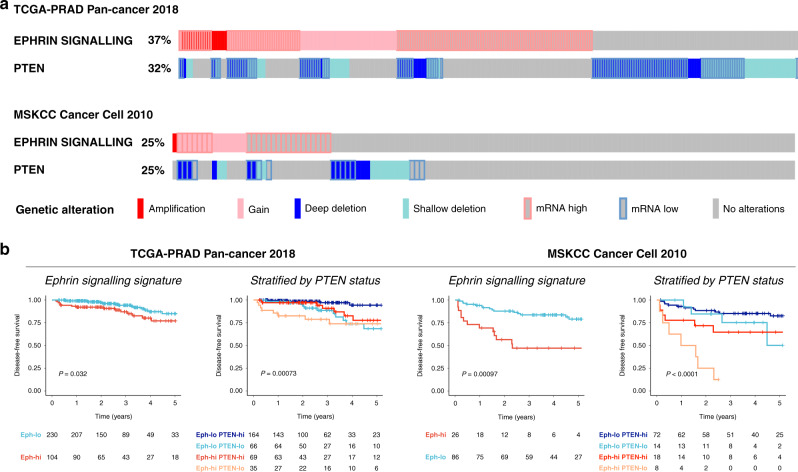
Upregulated Ephrin signalling in PTEN-deficient PCa correlates with poor disease-free survival. **a** cBioportal oncoprints of upregulated Ephrin signalling and PTEN status in TCGA-PRAD (*n* = 494); and MSKCC prostate cancer cohorts (*n* = 240). **b** Kaplan–Meier disease-free survival analysis for patients with available survival data in TCGA-PRAD (*n* = 334) and MSKCC (*n* = 112) cohorts stratified by either Ephrin signalling alone or in combination with PTEN status.

We have previously [16, 18] demonstrated a link between Ephrin signalling via pAkt^S473^ mediated phosphorylation and AA-induced bone marrow transendothelial invasion in PCa contributing to cellular migration and the metastatic phenotype. Interrogation of the TCGA-PRAD [36] and MSKCC Prostate Oncogenome Project [37] with an Ephrin signalling signature [38] showed upregulation of ephrin signalling in 37% and 25% of the cohorts, respectively (Fig. 1a), and this phenomenon was associated with a significant reduction in disease-free survival across both cohorts. Since pAkt activates Ephrin signalling, we measured the association between Ephrin signalling and activated Pi3k/Akt signalling, by sub-stratifying cohorts based upon PTEN status. Significant reduction in disease-free survival was observed amongst PCa patients with upregulated Ephrin signalling and PTEN-deficiency as compared with PCa patients with low Ephrin signalling and PTEN expression (univariate HR 8.43; 95% CI 2.53–28.05; *P* = 0.001 and HR 9.93; 95% CI 3.82–25.82; *P* < 0.001 for TCGA-PRAD and MSKCC datasets respectively (Fig. 1b and Supplementary Table 3). Thus, our data suggest that upregulated Ephrin signalling is associated with accelerated disease progression in patients with PTEN-deficient PCa.

### Arachidonic acid-induced bone marrow transendothelial migration requires pAkt^S473^ phosphorylation of EphA2 at serine 897

**Fig. 2:**
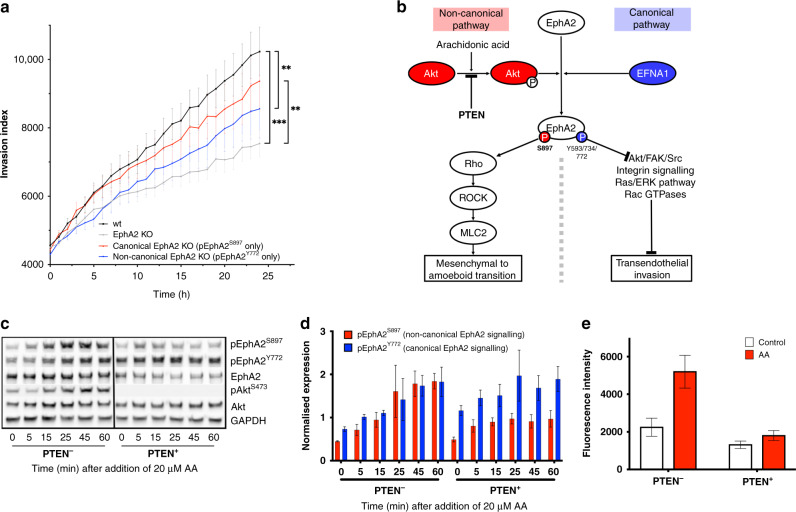
Arachidonic acid-induced transendothelial invasion requires pAkt dependent non-canonical pEphA2^S897^ signalling. **a** Real-time invasion assay towards a 20 µM AA substrate at 37 °C 5% CO_2_ through a Matrigel and BMEC barrier. **b** Diagrammatic representation of the non-canonical pathway initiated by arachidonic acid. **c** Western blot images showing temporal alteration in expression of total pEphA2^S897^, pEphA2^Y772^, EphA2, pAkt^S473^ and Akt after stimulation with 20 µM AA. **d** Temporal expression of pEphA2^Y772^ (canonical signalling) and pEphA2^S897^ (non-canonical signalling) in PTEN^−^ and PTEN^+^ PC3 cells after stimulation with 20 µM AA. **e** Endpoint invasion assay measuring invasion of wild-type PC3-PTEN^−^ or PC3-PTEN^+^ cells through a Matrigel and BMEC barrier towards 20 µM AA. Fluorescence was captured after 18-h incubation at 37 °C, 5% CO_2_.

Since aberrant Ephrin signalling adversely impacted disease-free survival amongst patients with PTEN-deficient PCa (Fig. 1), we sought to determine the potential mechanistic basis for this observation. Previously, our studies have shown that AA induced PCa metastasis through the non-canonical activation of EphA2 via pAkt^S473^ phosphorylation of EphA2^S897^ [16, 18]. We generated canonical EphA2^D739N^ and non-canonical EphA2^ΔS897G^ knockout mutants to model the effect of AA on kinetics of canonical and non-canonical EphA2 activity (Supplementary Fig. 1A, B). As expected, the loss of canonical signalling in the canonical kinase defective mutant EphA2^D739N^ did not significantly reduce PC3-M invasion through a BMEC/Matrigel barrier towards 20 µM AA. However, abrogated non-canonical activity (EphA2^ΔS897G^) significantly suppressed invasion (Fig. 2a). As non-canonical EphA2 activation requires phosphorylation of EphA2 at serine 897 by pAkt^S473^ (Fig. 2b), we sought to determine the effect of PTEN-mediated Akt regulation on invasion. Induction of PTEN expression in PC3 cells using a doxycycline-inducible PC3 cell line, reduced AA-mediated invasion to background levels (Fig. 2c, d) confirming the requirement of non-canonical EphA2 signalling for transendothelial invasion.

In a PTEN-deficient background, stimulation of PC3 cells with AA-induced both non-canonical pEphA2^S897^ signalling and canonical pEphA2^Y772^ signalling at similar levels. However, in the presence of PTEN, with subsequent reduction of pAkt^S473^, 20 µM AA predominantly induced canonical pEphA2^Y772^ signalling with limited induction of non-canonical pEphA2^S897^ (2:1 expression ratio) (Fig. 2c, d). This was accompanied with a loss of AA-induced invasion in the presence of PTEN (Fig. 2e).

### EphA2 overexpression is an independent prognostic marker in PTEN-deficient prostate cancer

**Fig. 3:**
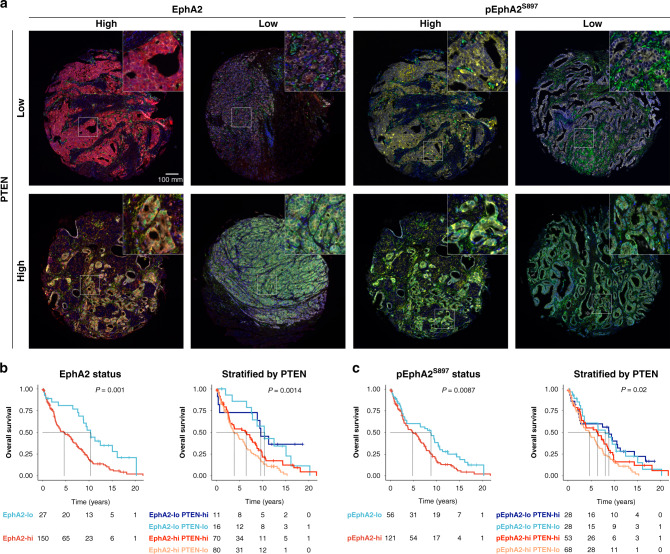
EphA2^hi^ and pEphA2^S897hi^ status are associated with poor overall survival in an unselected PCa TMA cohort. **a** Composite multiplex fluorescence images of representative TMA cores based upon EphA2 (red), pEphA2^S897^ (yellow) and PTEN (green) expression status. **b** Kaplan–Meier overall survival curves stratified by either EphA2 status alone or EphA2 and PTEN status. **c** Kaplan–Meier overall survival curves stratified by either pEphA2^S897^ status alone or pEphA2^S897^ and PTEN status. The black dotted line represents median survival. *P* value denotes the result of the log-rank test.

Since Ephrin signalling in PTEN-deficient PCa was associated with adverse outcome, which our in vitro data suggested was mediated by non-canonical activation via pEphA2^S897^, we sought to evaluate the influence of the upregulation of EphA2 markers on long-term overall survival using a PCa TMA cohort (*n* = 177) (patient demographics shown in Supplementary Data Table 2). PCa TMA sections were labelled with EphA2, pEphA2^S897^, PTEN, pan-cytokeratin, and a marker of MAT (Fig. 3a) and patients were categorised into high or low mean expression subgroups using maximally selected rank statistics.

Survival data and marker expression data were available for 177 patients with median follow-up of 16.5 years (95% CI 12.0–18.7). Upon multivariate analysis, high expression of both EphA2 and pEphA2^S897^ correlated with poor overall survival (Fig. 3b, c) with a median reduction in OS of 5.7 years (HR 2.34; 95% CI 1.25–4.39; *P* = 0.008) and 4.0 years (HR 1.63; 95% CI 1.05–2.54; *P* = 0.03), respectively. Disease characteristics of patients included in the TMA stratified by each marker are provided in Supplementary Data Table 3. As observed in vitro (Fig. 2), the activation of the non-canonical EphA2 pathway requires phosphorylation of EphA2^S897^ by pAkt^S473^, regulated by PTEN. Therefore, we sought to determine the potential of EphA2 status and the activation of the non-canonical EphA2 (pEphA2^S897^) signalling to sub-stratify patient outcome dichotomised by their PTEN status. Although the high expression of either EphA2 or pEphA2^S897^ adversely impacted overall survival in the whole cohort, these features were most prognostic amongst patients with PTEN^low^ PCa upon univariate Cox regression analysis (EphA2^hi^: HR 2.96, 95% 1.35–6.50, *P* = 0.007; pEphA2 ^S897hi^: HR 1.98, 95% CI 1.20–3.29, *P* = 0.008) (Fig. 3b, c and Table 1). Upon multivariate analysis, EphA2^hi^ PTEN^low^ patients had a significantly increased risk of poor overall survival compared to the reference EphA2^low^ PTEN^hi^ cohort (HR 3.13, 95% CI 1.11–8.82, *P* = 0.031).

**Table 1:** Overall survival stratified by EphA2, pEphA2^S897^ and PTEN expression in the Salford TMA cohort.

Overall survival	*N*	Median survival (years, 95% CI)	HR (univariable, 95% CI)	HR (multivariable, 95% CI)
EphA2 status				
Low	27	10.3 (8.8–NA)	Ref.	Ref.
High	150	4.6 (3.3–6.4)	2.22 (1.36–3.62, *P* = 0.001)	2.34 (1.25–4.39, *P* = 0.008)
pEphA2^S897^ status				
Low	56	8.8 (3.6–10.6)	Ref.	Ref.
High	121	4.8 (3.4–6.5)	1.62 (1.13–2.32, *P* = 0.009)	1.63 (1.05–2.54, *P* = 0.030)
EphA2/PTEN status				
EphA2^lo^ PTEN^hi^	11	9.6 (8.8-NA)	Ref.	Ref.
EphA2^lo^ PTEN^lo^	16	10.4 (7.8-NA)	1.18 (0.46–3.00, *P* = 0.734)	1.36 (0.44–4.22, *P* = 0.592)
EphA2^hi^ PTEN^hi^	70	6.4 (2.9–8.6)	2.05 (0.93–4.51, *P* = 0.076)	2.70 (0.98–7.45, *P* = 0.055)
EphA2^hi^ PTEN^lo^	80	3.8 (2.9–6.1)	2.96 (1.35–6.50, *P* = 0.007)	3.13 (1.11–8.82, *P* = 0.031)
pEphA2^S897^/PTEN status				
pEphA2^S897lo^ PTEN^hi^	28	8.8 (2.5–14.4)	Ref.	Ref.
pEphA2^S897lo^ PTEN^lo^	28	8 (3.5–12.5)	1.13 (0.62–2.07, *P* = 0.686)	1.22 (0.58–2.56, *P* = 0.601)
pEphA2^S897hi^ PTEN^hi^	53	6.4 (3.4–8.6)	1.44 (0.84–2.46, *P* = 0.181)	1.83 (0.94–3.57, *P* = 0.075)
pEphA2^S897hi^ PTEN^lo^	68	4.6 (2.9–6.3)	1.98 (1.20–3.29, *P* = 0.008)	1.80 (0.96–3.38, *P* = 0.066)

The number of patients, median survival, univariate and multivariate hazard ratios were reported, with 95% confidence interval (CI). Ref. denotes reference cohort.

### Expression of EphA2, pEphA2^S897^ and PTEN within tumour lesions is spatially related

Intra-tumoural heterogeneity poses a major challenge in the development of prognostic markers of most cancers [39]. For instance, Cyll et al. [40] noted heterogeneous PTEN expression amongst tumour lesions from 75% of radical prostatectomy samples. Therefore, we posited that expression of EphA2 and pEphA2 may display similar intra-tumoural heterogeneity and tested whether this phenomenon was associated also with the MAT process required for PCa transendothelial invasion [16].

**Fig. 4:**
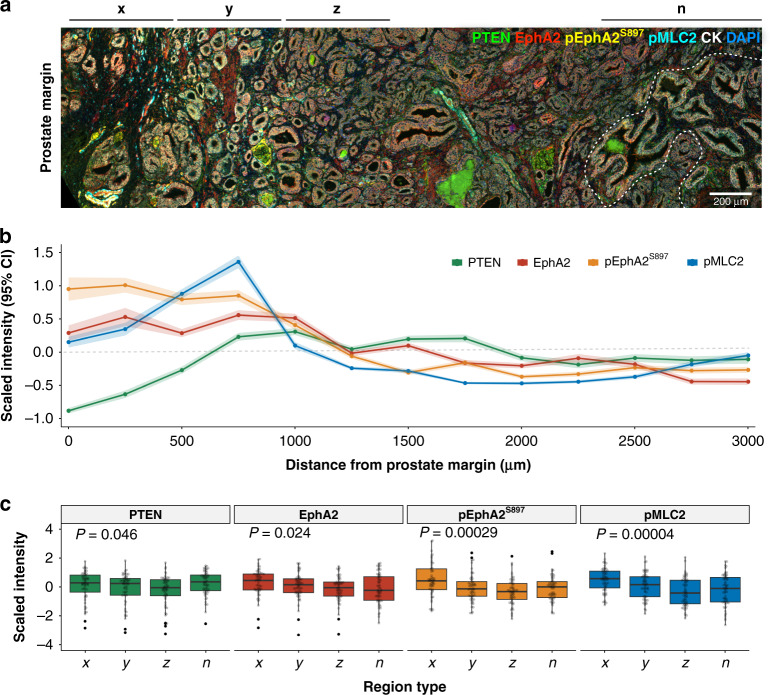
EphA2, pEphA2^S897^ and pMLC2 expression are spatially regulated within tumour lesions. **a** Representative multispectral image of a radical prostatectomy section stained for PTEN (green), EphA2 (red), pEphA2^S897^ (gold), pMLC2 (cyan), Cytokeratin (white) and DAPI (blue). Zone *x, y* and *z* are serial 500-µm wide zones from the prostate margin. A region of normal-adjacent glandular architecture (n) has been defined by the dotted white line. **b** Scaled intensity (95% confidence interval) of marker expression across the tissue section depicted in **a**. **c** Scaled intensity marker expression within each zone for PTEN, EphA2, pEphA2^S897^ and pMLC2 for the radical prostate cohort (*n* = 67). *P* values denote the result of Kruskal–Wallis test.

Expression of PTEN, EphA2, pEphA2^S897^ and the MAT marker pMLC2 was assessed in a radical prostatectomy cohort (*n* = 67), which provided access to larger sections of the prostate rather than biopsy cores, by multispectral immunofluorescence. Figure 4a shows a representative multispectral image of a prostate section labelled for PTEN, EphA2, pEphA2^S897^ and pMLC2 displaying intra-tumoural spatial heterogeneity. Quantitative assessment of marker expression (Fig. 4b) demonstrates EphA2 and pEphA2^S897^ were most highly expressed in the 500 µm closest to the prostate margin (zone *x*), decreasing rapidly on measurement deeper into the tumour (zones *y & z*), and eventually approaching levels comparable to an adjacent region of normal glandular tissue. In contrast, in this example, there is a loss of PTEN expression within zone *x*, with PTEN expression rising within zone *y* (500–1000 µm from prostate margin) and being maintained throughout the remainder of the tumour lesion.

Although there is variation between patients within this cohort, the global trends in marker expression within tumour loci show a clear pattern of spatial regulation relating to the distance from the prostate margin (Fig. 4c). Regions of the tumour within 500 µm of the prostate margin have increased expression of EphA2, pEphA2^S897^ and pMLC2, indicative of aggressive invasive MAT [16, 18] as compared with regions of adjacent normal glandular architecture. Expression significantly decreases within the tumour with expression levels comparable to normal glandular architecture observed within malignant cells at >1000 µm from the prostate margin.

## Discussion

Metastatic spread is a multistep process, often complicated by the dissemination to multiple sites. PCa preferentially metastasises to the axial skeleton, in particular the red bone marrow, often with subsequent visceral spread as tumour burden increases. This preferential metastatic process has enabled the generation of in vitro human bone metastatic models [35, 41, 42], allowing key pathways associated with bone metastatic behaviour to be uncoupled and characterised. Combining knowledge of these pathways with large tissue microarrays (TMA) that have long-term clinical PCa-specific survival data is a powerful approach enabling mechanistic study with a view to identifying new biomarkers and potential therapeutic targets.

Using this “bottom-up” methodology, we have shown previously that a crucial step in the initiation of bone metastatic propagation is the ability of malignant prostate epithelial cells to cross the endothelial blood-bone marrow stromal barrier [42]. Once within the bone marrow stroma the malignant prostate epithelial cells migrate towards and take up lipid from the bone marrow adipocytes, following which their morphology changes, assuming an aggressive and migratory phenotype [13–15, 43]. This observation led to the understanding that lipid signalling, in particular the ω-6 PUFA AA, is a key signalling pathway stimulating malignant prostate epithelial cells to migrate towards the red bone marrow. AA signalling induces the necessary mesenchymal to amoeboid transition of migrating prostate cancer cells required for transendothelial invasion and entry into the bone marrow stromal compartment [15, 16, 18]. AA-induced amoeboid transition requires the non-canonical activation of EphA2 via the phosphorylation of EphA2 at serine 897 by either pAkt or RSK [26, 27].

EphA2 has been shown to be a prognostic marker in a number of solid organ cancers [44] including breast [22], colorectal [45] and melanoma [46], and has been associated with adverse pathological features in PCa [47]. However, EphA2 can act as either a tumour suppressor or driver depending on the mode of activation. In this study, we assessed specifically and for the first time the expression of the ligand-independent non-canonical pEphA2^S897^ signalling known to drive AA-induced invasion in PCa. As non-canonical activation requires phosphorylation by pAkt, which is itself regulated by PTEN, a protein often lost during PCa progression, we assessed total EphA2 and pEphA2^S897^ expression alongside PTEN status.

We have shown previously, using an ω-6 PUFA arachidonic acid model, that MAT is required for PCa transendothelial invasion across a bone marrow endothelium and is initiated through the non-canonical EphA2 signalling pathway [16, 18]. We, therefore, hypothesised that the prognostic potential of pEphA2^S897^ for overall PCa survival would be greater than that of total EphA2 and that prognostic potential would be increased in the absence of the pAkt regulator PTEN. Within our TMA cohort, total EphA2 expression had a greater prognostic potential for overall survival than pEphA2^S897^, even in the PTEN^low^ cohort. Using our in vitro PTEN-deficient PC3 model of bone marrow invasion [35] we found that the ω-6 PUFA arachidonic acid (AA), a potent stimulator of MAT and bone marrow transendothelial invasion [15, 16], induced both canonical (Y772) and non-conical (S897) phosphorylation of EphA2. As expected, upon the introduction of PTEN, phosphorylation of EphA2 at S897 was reduced with subsequent inhibition of AA-induced invasion through a bone marrow endothelial barrier. However, there was no significant effect on AA-induced canonical EphA2 signalling via the phosphorylation of EphA2 at Y772 (Fig. 2). Taken together, our in vitro data suggest that whilst the non-canonical phosphorylation of EphA2 at S897 is required for AA-induced MAT, enabling malignant prostate epithelial cells to cross the bone marrow endothelium and migrate into the bone marrow, there is an induction of both canonical and non-canonical phosphorylation of EphA2. However, the increase in non-canonical phosphorylation of EphA2 and the reduction of juxtacrine signalling due to AA-induced MAT leads to increased expression of EphA2 through the reduction of EphA2 receptor recycling (reviewed in Cioce and Fazio [19]). This impact of increased EphA2 expression was observed also in our TMA, with significantly reduced overall survival amongst patients with high EphA2 expression.

High expression of both EphA2 and pEphA2^S897^ were shown to be a prognostic marker for poorer overall survival in both univariate and multivariate analysis. High EphA2 expression has been shown to be an independent prognostic marker for biochemical recurrence in the post-surgical setting [48]. Lin et al. [49] reported a correlation of PCa stage with EphA2 expression but our study is the first to report the prognostic potential of EphA2^hi^/PTEN^low^, showing substantially reduced overall survival when this phenotype is observed in diagnostic tissue specimens. The prognostic potential is strengthened in patients with PTEN-deficient PCa, where high EphA2 or pEphA2^S897^ expression was associated with a significant reduction in overall survival. This matched in silico findings from PTEN-stratified TCGA-PRAD and MSKCC cohorts using the Ephrin signalling signature derived in lung adenocarcinoma [38].

The potential use of tissue-based biomarkers is complicated further by intra-tumoural heterogeneity. Within this study, we found that expression of EphA2 and pEphA2^S897^ was spatially regulated within tumour lesions (Fig. 4). The highest expression of EphA2 and pEphA2^S897^ was observed in malignant cells within 500 µm of the prostate margin and expression declined with increasing distance from that margin. However, whilst invasive stimuli such as AA, only induce the non-canonical phosphorylation of EphA2 at the leading/invasive edge of the PCa lesion, we can detect an increased expression of total EphA2 deeper within the tumour lesion: this also correlated with poorer overall survival. This suggests that changes in expression of EphA2 itself could be of potential benefit as a prognostic marker using diagnostic tissue specimens, supporting previous findings that elevated EphA2 expression was associated with increased risk of treatment failure, e.g. following radical prostatectomy [48]. This observed phenomenon is also a potentially important observation for pathologists in their analysis and interpretation of prostate tissue samples, where, on the balance of evidence presented herein, greater emphasis needs to be given to closer analysis and grading of the leading edge/margin of the tumour itself.

The addition of clustered Ephrin-A1, in the form of Ephrin-A1 conjugated to an Fc receptor, can inhibit the induction of pEphA2^S897^ non-canonical signalling with subsequent internalisation and degradation of the EphA2 receptor [17, 18]. Although the use of natural Ephrin-A1 ligand is hampered by the lack of specificity between Ephrin ligands and receptors, the development of clustered EphA2-specific peptide agonists raises the potential for new therapeutic approaches [32]. In Fig. 2, we demonstrated that although PTEN status affects the levels of pEphA2^S897^ induced by AA, it had no effect on the induction of canonical EphA2 phosphorylation at Y772. This would suggest that the clinical potential of specific EphA2 agonists would be independent of PCa PTEN status. The induction of canonical EphA2 signalling would block the non-canonical pEphA2^S897^ signalling required for AA-induced transendothelial invasion of the bone marrow stroma and thereby potentially reduce the spread of malignant prostate epithelial cells to the bone marrow stromal compartment.

It should be noted that this is a single-centre study, albeit a study of archival tissue collected over a 12-year period and with a minimum of 15 years of clinical follow-up. Future work is therefore required to assess the biopotential in a larger multicentre trial of archival tissue before taking forward into a prospective study correlating non-canonical signalling at the point of diagnosis with clinical parameters such as Gleason Grade group, clinical-stage, genomic and imaging as a prognostic indicator for clinical outcome.

In summary, we have shown loss of PTEN regulation of pAkt enables AA to initiate non-canonical EphA2 signalling via pEphA2^S897^, with subsequent MAT, a cellular reconfiguration which is essential for bone marrow transendothelial invasion in vitro. Using a TMA of archival primary prostate cancer tissue linked to long-term clinical outcome data we have shown that EphA2 and pEphA2^S897^ are associated with substantially worse long-term survival in PTEN-deficient prostate cancer and that the migrational stimulatory effect is most pronounced at the tumour margin.

## Supplementary information

Supplementary Information

Supplementary Data File

aj-Checklist
