# Supplementary material for: Non-canonical EphA2 activation underpins PTEN-mediated metastatic migration and poor clinical outcome in prostate cancer
Source: Br J Cancer. 2022 Jul 22;127(7):1254–62. doi: 10.1038/s41416-022-01914-3 (PMC9519535; doi:10.1038/s41416-022-01914-3)
Supplement: Supplementary file 1 — Supplementary Information [file 41416_2022_1914_MOESM1_ESM.docx]

**Supplementary Information**

**Detailed Methodology**

**Antibodies:**

| **Antibody Name** | **Use** | **Source** |
| --- | --- | --- |
| pEphA2^S897^ (D9A1) (6347S) | WB/IF | Cell Signalling Technologies (Hitchin, UK) |
| pEphA2^Y772^ (8244S) | WB | Cell Signalling Technologies (Hitchin, UK) |
| pAkt^S473^ (193H12) (4058S) | WB | Cell Signalling Technologies (Hitchin, UK) |
| Akt (9272S) | WB | Cell Signalling Technologies (Hitchin, UK) |
| PTEN (D4.3) (9188S) | IF | Cell Signalling Technologies (Hitchin, UK) |
| pMLC2 (3674S | IF | Cell Signalling Technologies (Hitchin, UK) |
| Pan-cytokeratin (C11) (C2931) | IF | Sigma-Aldrich / Merck, Poole, UK |
| DAPI (D9542) | IF | Sigma-Aldrich / Merck, Poole, UK |
| EphA2 clone D7 (05-480) | WB | Merck / Millipore (Watford, UK) |
| GAPDH antibody (ab9485) | WB | Abcam (Cambridge, UK). |
| EphA2 (C-3) (sc-398832) | IF | Santa Cruz / Insight Biotechnology, Wembley, UK |
| Tyramide Signal Amplification (TSA) Opal 520, Opal 620, Opal 570, Opal 690 Reagent Packs | IF | Akoya Biosciences Inc., Marlborough, MA 01752, USA |
|  |  |  |

**Supplementary table 1.** Antibodies use and source. IF Immunofluorescence. WB Western blot.

**Multiplex Immunofluorescence**

TMA PCa tissue cores and whole mount prostate sections were identified and graded by consultant genitourinary histopathologists (JS/PO) using the 2014 Grading Group criteria (49). Normal-adjacent tissue cores were identified where available. Freshly cut 4 μm TMA sections were mounted on slides, dewaxed and heat induced epitope retrieval was performed using a buffer of TRIS/EDTA, pH 9.0. pEphA2^S897^ antibody was placed on the slides overnight at 4°C at a concentration of 1:100 dilution. The slides were washed with PBS, blocked with 0.3% H_2_O_2_ for 10 minutes and wet loaded onto the Ventana Discovery Ultra automated IHC/ISH research platform. Following an HRP conjugation step, a TSA Opal 620 1:100 was linked to the pEphA2^S897^ antibody previously bound to the slide overnight. After the heat denaturation step the slides were washed and a second primary antibody EphA2 (C-3) was placed on the slides overnight at 4°C at a concentration of 4 µg/mL. The following morning slides were washed with PBS and wet loaded onto the Ventana Discovery Ultra automated IHC/ISH research platform where the HRP conjugate was applied followed by TSA Opal 570 1:150. All further primary and secondary antibodies were bound to the slides and stained in a fully automated manner in the following order with a heat denaturation step at 95°C for 8 min between each antibody cycle: PTEN (D4.3) 1:50 / TSA Opal 520 1:100; pan cytokeratin (C11) 1:10,000 / TSA Opal 690 1:150; DAPI. Slides were mounted in ProLong Gold antifade reagent (Invitrogen, Paisley, UK) and scanned on an Akoya Biosciences Vectra 3 system using the x20 magnification lens.

**Data analysis**

TMA image analysis was performed using InForm v2.4 (Akoya Biosciences Inc., Marlborough, MA 01752, USA). Operator screening was used to provide quality control for the automated core detection and region of interest (ROI) identification. Data for 270 patients were available for analysis. Segmented single cell data were analysed for 177 patients from the TMA who had complete clinical outcome data on RStudio v1.1.423.

Radical prostatectomy tissue sections were labelled for multiplex immunofluorescence and imaged using the same protocol used for TMA tissue sections. However, single cell analysis was performed in HALO v3.0.311.299 (Indica Labs, Albuquerque, USA) image analysis software with downstream analysis performed in RStudio v1.1.423 as indicated above.

**Antigen preservation in archival prostate tissue**

Using radical prostatectomy samples, we evaluated spatial heterogeneity in marker expression. To confirm that the differential expression of markers was spatially related and not an artefact of poor antigen preservation deeper within the tissue, we assessed the expression of each marker individually over the entire radical prostatectomy section. Supplementary figure 2 shows the single channel expression of each marker in greyscale for the representative radical prostatectomy section shown in figure 4.

Pan cytokeratin expression is uniform across the whole tissue section. EphA2, pEphA2^S897^ and pMLC2 show differential expression, with tumour tissue deeper within the organ demonstrating reduced expression of these markers. This spatial alteration in marker expression is unlikely to be due to poor antigen preservation. The expression within non-malignant tissue shows uniform staining for all markers across the radical prostatectomy sections. The ROIs highlighted in the EphA2 and pMLC2 sections also show areas of strong and discrete staining of non-malignant tissue. This demonstrates that antigens were preserved within the radical prostatectomy sections used within this study.

| **Patient characteristics** | **Overall (%)** |
| --- | --- |
| **Age at diagnosis** |  |
| Median (IQR) | 73.0 (10.0) |
| <65 | 30 (16.9) |
| 65-74 | 60 (33.9) |
| >74 | 81 (45.8) |
| (Missing) | 6 (3.4) |
|  |  |
| **PSA at diagnosis** |  |
| Median (IQR) | 34.0 (88.5) |
| 0-9.9 | 33 (18.6) |
| 10-19.9 | 30 (16.9) |
| 20-99.9 | 66 (37.3) |
| >=100 | 45 (25.4) |
| (Missing) | 3 (1.7) |
|  |  |
| **Grade Group** |  |
| GG 1-2 | 102 (57.6) |
| GG 3 | 19 (10.7) |
| GG 4-5 | 28 (15.8) |
| (Missing) | 28 (15.8) |
|  |  |
| **Clinical tumour stage** |  |
| T1-T2 | 68 (38.4) |
| T3 | 63 (35.6) |
| T4 | 37 (20.9) |
| Tx | 9 (5.1) |
|  |  |
| **M stage at diagnosis** |  |
| M0 | 76 (42.9) |
| M1 | 51 (28.8) |
| Mx | 50 (28.2) |
|  |  |
| **Risk group** |  |
| Low | 16 (9.0) |
| Intermediate | 25 (14.1) |
| High | 64 (36.2) |
| Metastatic | 72 (40.7) |

**Supplementary Table 2. Patient demographics of the Salford PCa TMA**


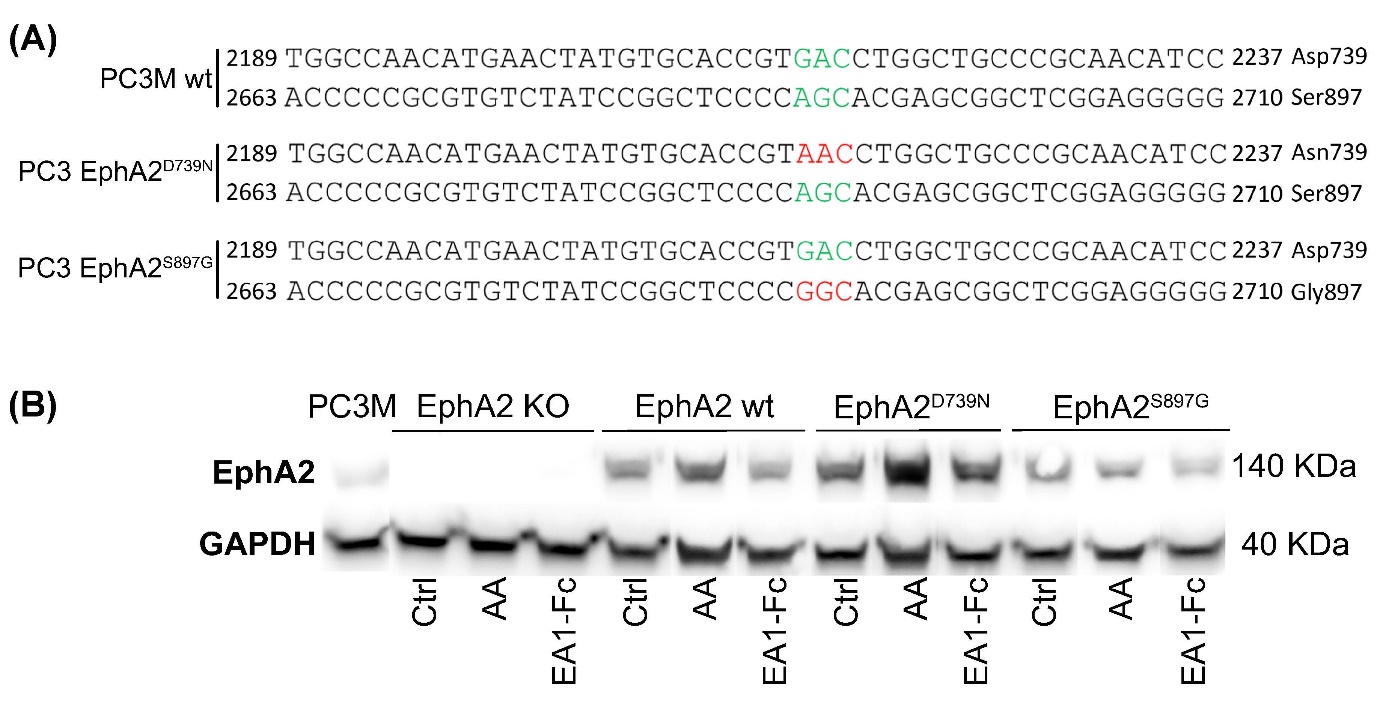


**Supplementary Figure 1. Arachidonic acid induced transendothelial invasion requires pAkt dependent non-canonical pEphA2^S897^ signalling.** A) EphA2 sequence showing site directed mutagenesis of phosphorylation sites at D739 and S897. B) Composite western blot confirmation of EphA2 expression in PC3-M mutants


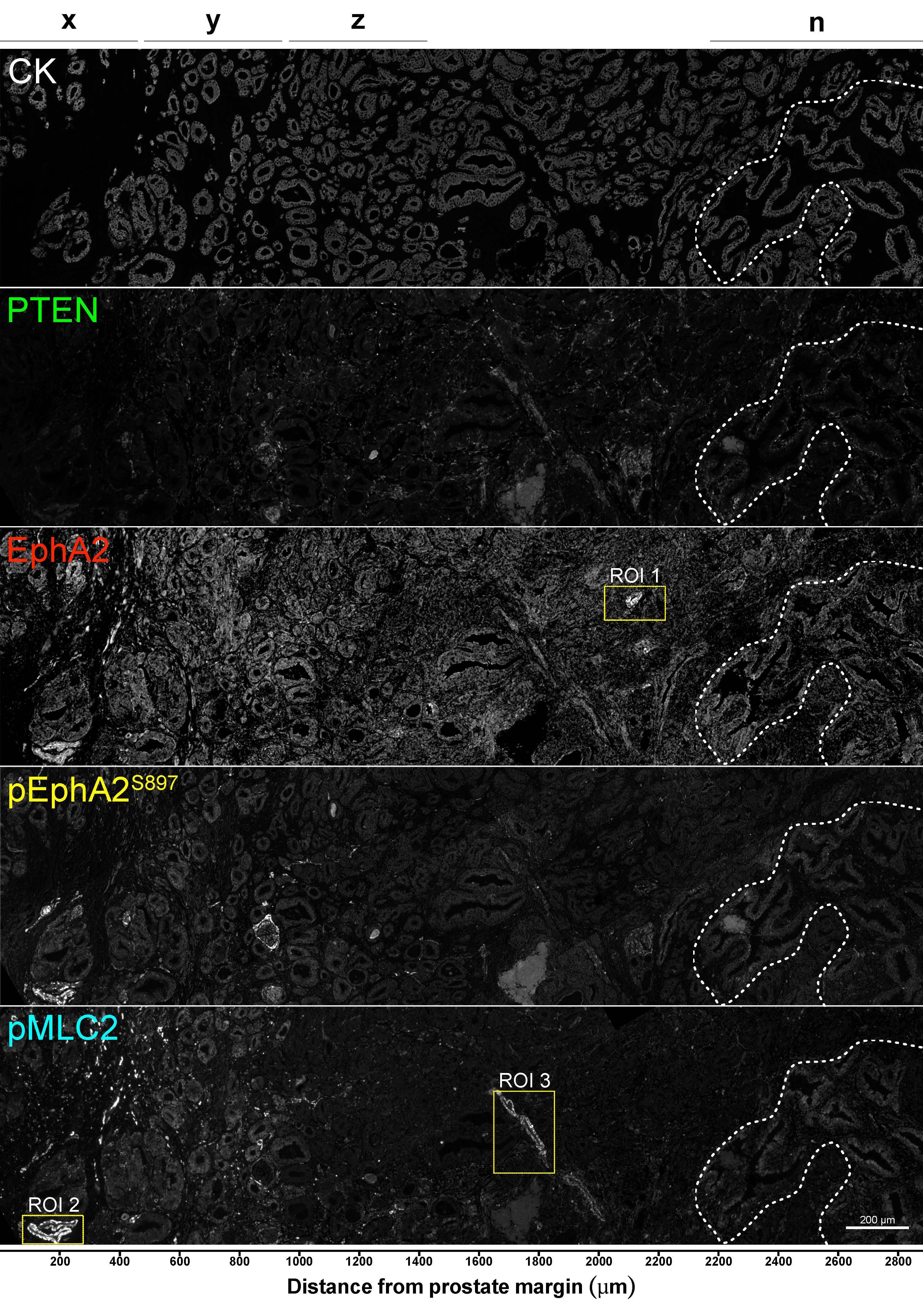

**Supplementary Figure 2.** **Antigen preservation within radical prostatectomy sections. EphA2, pEphA2^S897^ and pMLC2 expression is spatially regulated within tumour lesions.** Single channel images displayed in greyscale used to generate the multispectral image of a radical prostatectomy section in figure 4. Each panel is labelled with the marker and the colour of this marker within figure 4. Spatial zones X, Y and Z are shown representing sequential 500 µm zones from the prostate margin and a region of normal architecture is outlined (n). ROI = Region of Interest.


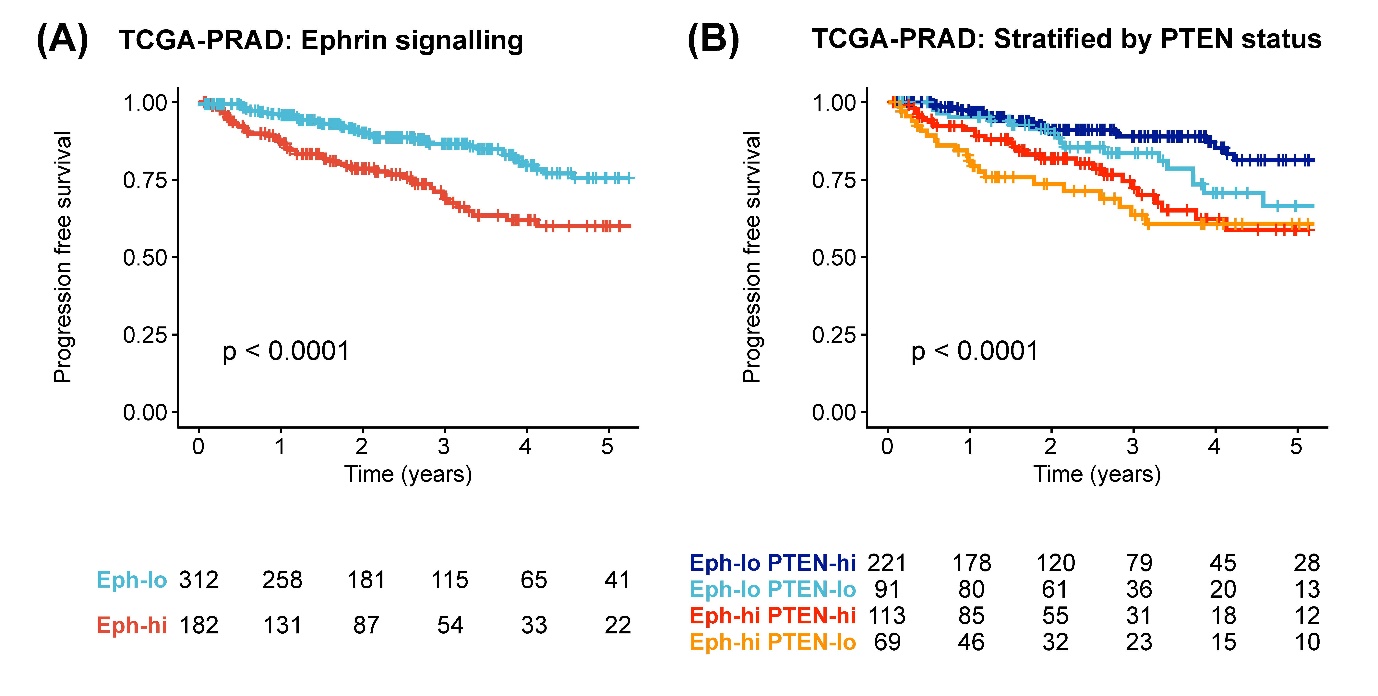


**Supplementary Figure 3. Upregulated Ephrin signalling in PTEN deficient prostate cancer correlates with poor progression-free survival.** Kaplan-Meier progression-free survival analysis for patients with available survival data in TCGA-PRAD (n=494) (36) cohort stratified by either Ephrin signalling alone or in combination with PTEN status.

|  | **Category** | **N (%)** | **HR (univariate, 95% CI)** | **HR (multivariate, 95% CI)*** |
| --- | --- | --- | --- | --- |
| **Disease-free survival (DFS)** | |  |  |  |
| **TCGA-PRAD** | Eph-lo PTEN-hi | 164 (49.1) | Ref. | Ref. |
|  | Eph-lo PTEN-lo | 66 (19.8) | 5.96 (1.90-18.74, p=0.002) | 4.27 (1.31-13.86, p=0.016) |
|  | Eph-hi PTEN-hi | 69 (20.7) | 3.96 (1.16-13.55, p=0.028) | 3.22 (0.93-11.16, p=0.066) |
|  | Eph-hi PTEN-lo | 35 (10.5) | 8.43 (2.53-28.05, p=0.001) | 4.12 (1.16-14.64, p=0.028) |
| **MSKCC** | Eph-lo PTEN-hi | 72 (64.3) | Ref. | Ref. |
|  | Eph-lo PTEN-lo | 14 (12.5) | 1.76 (0.58-5.38, p=0.321) | 2.64 (0.78-9.00, p=0.120) |
|  | Eph-hi PTEN-hi | 18 (16.1) | 1.99 (0.77-5.14, p=0.156) | 1.68 (0.63-4.49, p=0.301) |
|  | Eph-hi PTEN-lo | 8 (7.1) | 9.93 (3.82-25.82, p<0.001) | 4.07 (1.26-13.12, p=0.019) |
| **Progression-free survival (PFS)** | | |  |  |
| **TCGA-PRAD** | Eph-lo PTEN-hi | 221 (44.7) | Ref. | Ref. |
|  | Eph-lo PTEN-lo | 91 (18.4) | 1.80 (0.98-3.30, p=0.058) | 1.25 (0.65-2.42, p=0.503) |
|  | Eph-hi PTEN-hi | 113 (22.9) | 2.57 (1.48-4.46, p=0.001) | 2.18 (1.22-3.88, p=0.008) |
|  | Eph-hi PTEN-lo | 69 (14.0) | 3.47 (1.95-6.19, p<0.001) | 1.81 (0.95-3.45, p=0.073) |
| *Adjusted for age at diagnosis, PSA at diagnosis, ISUP grade group, pathological tumour stage, and pathological nodal stage. | | | | |
|  |  |  |  |  |

**Supplementary Table 3. Disease free (TCGA-PRAD and MSKCC datasets) and progression free (TCGA-PRAD) survival.** Number of patients, univariate and multivariate hazard ratios reported, with 95% confidence interval (CI). Ref. denotes reference cohort.


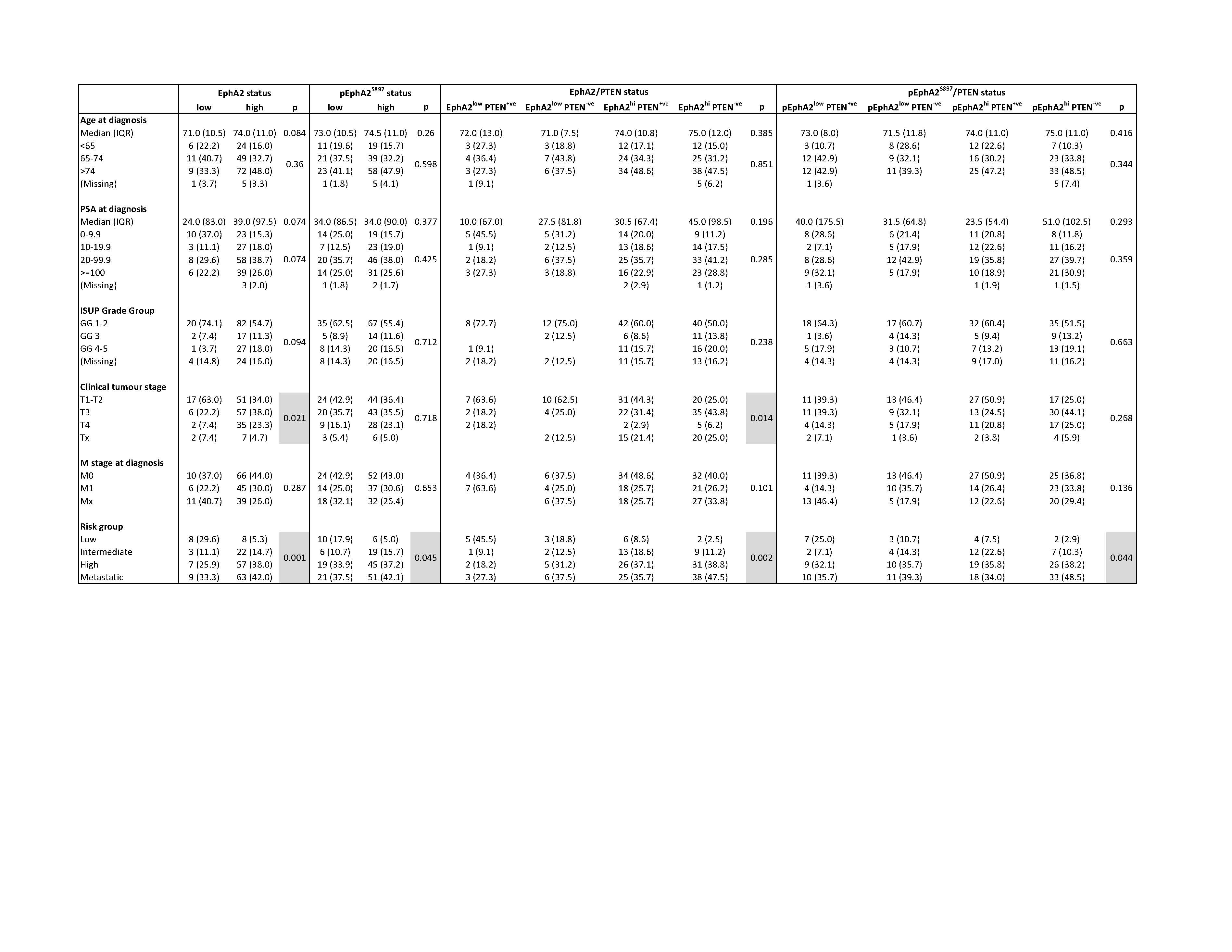


**Supplementary Table 4. Patient and disease characteristics of the Salford prostate Cancer TMA stratified according to marker status.**

|  | **HR (univariable)** | **HR (multivariable)** | | | |
| --- | --- | --- | --- | --- | --- |
|  |  | **EphA2 status** | **pEphA2^S897^ status** | **EphA2/PTEN status** | **pEphA2^S897^/PTEN status** |
| **Age at Diagnosis** |  |  |  |  |  |
| <65 | - | - | - | - | - |
| 65-74 | 0.99 (0.59-1.65, p=0.973) | 1.07 (0.56-2.02, p=0.841) | 1.24 (0.67-2.31, p=0.496) | 1.09 (0.57-2.09, p=0.786) | 1.28 (0.68-2.40, p=0.448) |
| >74 | 2.16 (1.31-3.54, p=0.002) | 2.67 (1.48-4.82, p=0.001) | 2.89 (1.60-5.23, p<0.001) | 2.74 (1.51-4.98, p=0.001) | 2.96 (1.62-5.41, p<0.001) |
|  |  |  |  |  |  |
| **PSA at Diagnosis** |  |  |  |  |  |
| 0-9.9 | - | - | - | - | - |
| 10-19.9 | 1.13 (0.63-2.03, p=0.688) | 0.77 (0.38-1.55, p=0.468) | 0.82 (0.41-1.66, p=0.588) | 0.77 (0.38-1.54, p=0.456) | 0.82 (0.41-1.66, p=0.583) |
| 20-99.9 | 1.91 (1.16-3.13, p=0.011) | 0.99 (0.53-1.85, p=0.975) | 1.12 (0.60-2.09, p=0.720) | 0.98 (0.52-1.84, p=0.954) | 1.12 (0.60-2.08, p=0.727) |
| >=100 | 2.22 (1.32-3.73, p=0.003) | 1.19 (0.59-2.42, p=0.626) | 1.41 (0.69-2.86, p=0.344) | 1.21 (0.60-2.48, p=0.593) | 1.45 (0.70-3.00, p=0.311) |
|  |  |  |  |  |  |
| **Gleason Group** |  |  |  |  |  |
| GG 1-2 | - | - | - | - | - |
| GG 3 | 1.78 (1.03-3.07, p=0.040) | 1.56 (0.85-2.86, p=0.154) | 1.41 (0.77-2.61, p=0.269) | 1.53 (0.83-2.82, p=0.171) | 1.39 (0.75-2.58, p=0.296) |
| GG 4-5 | 2.72 (1.73-4.27, p<0.001) | 1.96 (1.21-3.19, p=0.007) | 2.20 (1.36-3.56, p=0.001) | 1.95 (1.20-3.17, p=0.007) | 2.20 (1.36-3.57, p=0.001) |
|  |  |  |  |  |  |
| **Clinical Stage** |  |  |  |  |  |
| T1-T2 | - | - | - | - | - |
| T3 | 1.99 (1.36-2.93, p<0.001) | 1.46 (0.87-2.47, p=0.154) | 1.58 (0.94-2.64, p=0.084) | 1.42 (0.83-2.42, p=0.200) | 1.61 (0.94-2.74, p=0.081) |
| T4 | 1.92 (1.22-3.02, p=0.005) | 1.27 (0.70-2.28, p=0.430) | 1.37 (0.76-2.47, p=0.288) | 1.20 (0.65-2.19, p=0.560) | 1.36 (0.75-2.49, p=0.311) |
| Tx | 6.89 (3.11-15.27, p<0.001) | 8.11 (2.54-25.84, p<0.001) | 8.50 (2.72-26.56, p<0.001) | 7.71 (2.38-25.04, p=0.001) | 8.47 (2.70-26.54, p<0.001) |
|  |  |  |  |  |  |
| **M Stage at Diagnosis** |  |  |  |  |  |
| M0 | - | - | - | - | - |
| M1 | 2.26 (1.52-3.37, p<0.001) | 2.01 (1.23-3.30, p=0.006) | 1.80 (1.11-2.94, p=0.018) | 1.98 (1.21-3.26, p=0.007) | 1.78 (1.09-2.91, p=0.022) |
| Mx | 1.23 (0.82-1.84, p=0.324) | 2.11 (1.25-3.55, p=0.005) | 2.10 (1.24-3.54, p=0.006) | 2.15 (1.27-3.65, p=0.004) | 2.18 (1.26-3.76, p=0.005) |

**Supplementary Table 5. Cox regression analysis by marker status of disease characteristics of the Salford prostate cancer TMA**
